# New Appliances and Things Medical

**Published:** 1895-04-06

**Authors:** 


### NEW APPLIANCES AND THINGS MEDICAL.

[We shall be glad to receive, at our Office, 428, Strand, London, W.C., from the manufacturers, specimens of all new preparations and appliances which may be brought out from time to time.]

### HOVIS BREAD AND BISCUITS.

(S. Fitton and Son, Macclesfield; and R. Middlemass and Son, Causewayside, Edinburgh.)

The term Hovis has been given to these preparations in order to designate that they have been manufactured from the Hovis flour. This flour is made by a patented process in such a way that the constituents of the germ or embryo of the wheat, which has to be removed from the flour in ordinary milling in order to render the flour free from the diastasic ferments which would impair its keeping qualities, and cause in some cases sourness in the bread manufactured from it, are restored to the flour. As the germ is especially rich in phosphates and nitrogenous foods, its removal from flour causes a diminution in the amount of these valuable constituents, and whole meal and brown bread have been recommended by food specialists on the ground that they have not been taken away. In the Hovis patent the germ is removed from the flour and heated in a special manner, so as to kill the ferments present in it, and then it is once more returned to the flour. Hovis flour, therefore, differs from that used in making brown bread, in containing no bran, and in having the germ in an inert condition. The absence of the bran renders the product free from horny and irritating particles, whilst the destruction of the vitality of the embryonic ferments ensures the absence of fermentation in the flour produced. In order to verify these statements, the bread and biscuits submitted to us were examined mechanically and chemically. The microscopic and mechanical examination revealed the fact that there was an entire absence of the fragments of bran, which are to be met with in brown bread and whole-meal bread, and thus showed that the bread and biscuits are free from any portions which might cause irritation to the mucous membrane and internal linings of the body. The chemical analysis of the bread and biscuit is summarised in the following table, the quantity of nitrogen found being returned as gluten :—

| | Bread. | Biscuit. |
|---|---|---|
| Moisture ... ... ... | 47·12 ... | 4·07 |
| Ash (chiefly phosphates) ... | 1·17 .. | 2·07 |
| Gluten... ... ... ... | 12·14 ... | 14·72 |
| Starch and fat (difference) ... | 39·57 ... | 79·50 |

It will be seen from the above analysis that the Hovis preparations have an exceptionally high amount of phosphates and nitrogenous substances, since in an ordinary flour containing about 12 per cent of moisture the ash averages about 0·5 per cent. and the quantity of gluten from 8 to 11 per cent. It is claimed for the Hovis food biscuits that they possess in the highest degree all the essentials of a perfect food, and our examination goes far to confirm the high opinion which the proprietors hold with regard to them. We ought to add that the flavour of the bread and biscuits has not been injured in any way by the treatment, but that they possess a most agreeable nutty taste.

